# Exenatide Treatment Alone Improves β-Cell Function in a Canine Model of Pre-Diabetes

**DOI:** 10.1371/journal.pone.0158703

**Published:** 2016-07-11

**Authors:** Viorica Ionut, Orison O. Woolcott, Hasmik J. Mkrtchyan, Darko Stefanovski, Morvarid Kabir, Malini S. Iyer, Huiwen Liu, Ana V. B. Castro, Qiang Wu, Josiane L. Broussard, Cathryn M. Kolka, Isaac Asare-Bediako, Richard N. Bergman

**Affiliations:** 1 Diabetes and Obesity Research Institute, Cedars-Sinai Medical Center, Los Angeles, California, United States of America; 2 Department of Physiology and Biophysics, University of Southern California, Los Angeles, California, United States of America; University of Catanzaro Magna Graecia, ITALY

## Abstract

**Background:**

Exenatide’s effects on glucose metabolism have been studied extensively in diabetes but not in pre-diabetes.

**Objective:**

We examined the chronic effects of exenatide alone on glucose metabolism in pre-diabetic canines.

**Design and Methods:**

After 10 weeks of high-fat diet (HFD), adult dogs received one injection of streptozotocin (STZ, 18.5 mg/kg). After induction of pre-diabetes, while maintained on HFD, animals were randomized to receive either exenatide (n = 7) or placebo (n = 7) for 12 weeks. β-Cell function was calculated from the intravenous glucose tolerance test (IVGTT, expressed as the acute insulin response, *AIR*_*G*_), the oral glucose tolerance test (OGTT, insulinogenic index) and the graded-hyperglycemic clamp (clamp insulinogenic index). Whole-body insulin sensitivity was assessed by the IVGTT. At the end of the study, pancreatic islets were isolated to assess β-cell function *in vitro*.

**Results:**

OGTT: STZ caused an increase in glycemia at 120 min by 22.0% (interquartile range, IQR, 31.5%) (P = 0.011). IVGTT: This protocol also showed a reduction in glucose tolerance by 48.8% (IQR, 36.9%) (P = 0.002). *AIR*_*G*_ decreased by 54.0% (IQR, 40.7%) (P = 0.010), leading to mild fasting hyperglycemia (P = 0.039). Exenatide, compared with placebo, decreased body weight (P<0.001) without altering food intake, fasting glycemia, insulinemia, glycated hemoglobin A1c, or glucose tolerance. Exenatide, compared with placebo, increased both OGTT- (P = 0.040) and clamp-based insulinogenic indexes (P = 0.016), improved insulin secretion *in vitro* (P = 0.041), but had no noticeable effect on insulin sensitivity (P = 0.405).

**Conclusions:**

In pre-diabetic canines, 12-week exenatide treatment improved β-cell function but not glucose tolerance or insulin sensitivity. These findings demonstrate partial beneficial metabolic effects of exenatide alone on an animal model of pre-diabetes.

## Introduction

Insulin resistance and β-cell dysfunction play fundamental roles in the pathogenesis of type 2 diabetes. Impairment of β-cell function to compensate to for insulin resistance accelerates the progression to type 2 diabetes [1].

Exenatide, a synthetic analogue of exendin-4, a natural ligand of the glucagon-like peptide 1 receptor, has been extensively used for type 2 diabetes treatment. Exenatide has been shown to reduce hyperglycemia, promote body weight loss, and improve insulin sensitivity and β-cell function, resulting in lower hemoglobin A1c levels [2]. However, it is unclear whether these multiple effects are related to exenatide *per se* or concomitant interventions such as lifestyle changes or combined antidiabetic drugs. Numerous studies have explored the therapeutic effects of exenatide in type 2 diabetes [3], but few studies have examined its metabolic effects on pre-diabetes. Impaired fasting glucose and impaired glucose tolerance are well established risk factors for type 2 diabetes [4–7]. Treatment of these pre-diabetic conditions has been associated with delayed progression to diabetes [6]. Previous clinical studies have explored the effect of exenatide on glucose tolerance (in combination with lifestyle changes) [8], insulin sensitivity, and β-cell function (in combination with pioglitazone and metformin) [9], and the homeostasis model assessment-insulin resistance index [10]. However, none of these studies has systematically explored the effect of exenatide alone on glucose homeostasis in the pre-diabetes state.

The prevalence of pre-diabetes (impaired fasting glucose or impaired glucose tolerance) in the United States has been estimated in ~35% [11]. Given the high prevalence of pre-diabetes, a high risk factor for type 2 diabetes [4], and the widely use of exenatide in the clinical practice, we thought it is relevant to further study the metabolic effects of exenatide alone in a canine model of pre-diabetes. In the present study, we hypothesize that chronic treatment with exenatide alone improves glucose homeostasis in the pre-diabetic state. Thus, we determined the effects of exenatide on glucose tolerance, β-cell function, and insulin sensitivity in a canine model of pre-diabetes.

## Materials and Methods

### Animals and diet regimen

Experiments were conducted in adult male mongrel dogs, 1–2 years old. Dogs were supplied by Antech, Inc. (Barnhart, MO). Animals were single housed in stainless steel kennels in the vivarium of the Keck School of Medicine, University of Southern California (Los Angeles, CA). Kennels had gates between runs, fiberglass slatted floors or plastic coated expanded metal floors (24 square feet of floor space), and stainless steel feeders. Animals were permitted social contact between the runs through a steel mesh wall. Animals were provided with environmental enrichment. Dogs received positive interactions with animal care staff on a daily basis. Dogs were exercised within the room during room cleaning. Before the commencement of the study, animals received a standard diet consisted of 825 g of dry chow and one canned food (Hill’s Pet Nutrition, Topeka, KS) for 2–3 weeks [12]. Thereafter, animals were fed a hypercaloric high-fat diet (HFD) until the end of the study. HFD diet consisted of 825 g of dry chow and one canned food supplemented with lard (6 g/kg of baseline body weight). Total daily food presented (09:00–12:00 h) contained 5,527 kcal (53.0% from fat). Daily food intake was assessed by subtracting the weight of food presented from the weight of food left in the bowl or dropped on the floor. Water was provided *ad libitum*.

### Study design

All *in vivo* experiments were performed in the morning, after 12–16 hours of fasting. Biopsies from liver and pancreas for *in vitro* experiments were obtained at the end of study, prior to euthanasia, under general inhalant anesthesia (3% isoflurane). The full study protocol was approved by the Institutional Animal Care and Use Committees from the University of Southern California and the Cedars-Sinai Medical Center (Los Angeles, CA).

After an initial 10 weeks of HFD, starting at week −15, animals received a single intravenous dose of streptozotocin (STZ, 18.5 mg/kg of body weight) at week −5. This dose was previously found to be effective in fat-fed canines to induce pre-diabetes [13]. Five weeks after STZ administration, animals were randomized to receive either exenatide (n = 7; 10 μg subcutaneously b.i.d. during the weekdays and once daily during weekends) or placebo (n = 7). Drug treatment started at week 0 and continued until the end of the study (week 12, our primary endpoint). HFD was initiated at week −15 and was maintained until the end of the study. The oral glucose tolerance test (OGTT) and the intravenous glucose tolerance test (IVGTT) were performed in all animals at the beginning of the study, prior to the commencement of exenatide treatment (week 0), and at the end of the study. Body weight was assessed weekly. Daily food intake was recorded starting at week 0. IVGTT was also performed at weeks 3, 6, and 9. Fasting plasma glucose and insulin were also measured during IVGTT experiments. Hyperglycemic clamp and magnetic resonance imaging (MRI) of the abdominal region were performed at weeks 0 and 12. Likewise, assessment of fasting plasma glucagon, glycated hemoglobin A1c, and energy expenditure (resting metabolic rate) were performed at weeks 0 and 12.

Exenatide was kindly provided by Amylin Pharmaceuticals Inc. Streptozotocin was purchased from Sigma-Aldrich (St Louis, MO), dissolved in citrate buffer solution (pH 4.5; Sigma-Aldrich) immediately before injection [13].

### Experimental procedures

#### MRI

Fat distribution in the abdominal region was assessed under general anesthesia by MRI using a 1.5-T Siemens Scanner [12]. MRI scan included eleven transverse 10-mm thick slices. For each slice, major regions of the abdominal anatomy (extra- and intra-abdominal compartments) were defined by manual drawing. Fat and non-fat tissues were distinguished based on pixel intensity using an imaging software (sliceOmatic 4.3, Tomovision, Magog, QC, Canada). Total abdominal fat volume included the sum of subcutaneous and visceral fat obtained from the 11 slices [12].

#### OGTT

Glucose 50% was given as a bolus by oral gavage. Blood samples were taken at 15, 30, 45, 60, 90, 120, and 180 min [14]. Glucose tolerance was measured as the glucose concentration at t = 120 min. β-Cell function was measured as the OGTT insulinogenic index: the ratio of the over-basal area under the curve (AUC) of plasma insulin (mU/L) to over-basal AUC of plasma glucose (mg/dL). AUCs of plasma glucose (AUC_GLUCOSE_), insulin (AUC_INSULIN_), and glucagon (AUC_GLUCAGON_) were calculated using the trapezoidal rule (Prism 4.0, GraphPad Software, Inc.).

#### IVGTT

β-Cell function (acute insulin response, *AIR*_*G*_), whole-body insulin sensitivity (*S*_*I*_), the disposition index (*DI*), glucose effectiveness (*S*_*G*_), intravenous glucose tolerance (*K*_*G*_), and hepatic insulin clearance were calculated from the IVGTT, performed as previously described [15, 16]. Hepatic insulin clearance was expressed as the fractional clearance rate of insulin (*FCR*) and the metabolic clearance rate of insulin (*MCR*) [17]. A bolus of glucose 50% (0.3 g/kg of body weight) was given intravenously at 0 min and a bolus of insulin (0.03 U/kg of body weight) at 20 min. Blood samples were taken at t = 2, 3, 4, 5, 6, 8, 10, 12, 14, 16, 19, 22, 23, 24, 25, 30, 40, 50, 60, 70, 80, 90, 100, 110, 120, 140, 160, and 180 min. *AIR*_*G*_, *S*_*I*_, *DI*, and *S*_*G*_ were calculated using MINMOD (Minmod Millennium version 6.02, MINMOD Inc., Los Angeles, CA). *K*_*G*_ was calculated as the negative slope of the natural log of glucose versus time from t = 10 to t = 19 min. *FCR* and *MCR* were calculated using WINSAAM (version 3.3.0, University of Pennsylvania) [17]. *FCR* was calculated from the decay of plasma insulin after the intravenous bolus. Insulin from t = 22–80 min was fit to the following exponential decay curve. *MCR* was calculated as the ratio of the dose of injected insulin during the IVGTT to the over-baseline area under the curve of insulin after time of injection to infinity.

#### Graded-hyperglycemic clamp

Glucose 50% was infused intravenously in a peripheral vein at variable rates so as to maintain blood glucose constant at three sequential concentrations: 100 mg/dL (*t* = 0–59 min), 150 mg/dL (*t* = 60–149 min) and 200 mg/dL (*t* = 150–240 min) [18]. β-Cell function was calculated as the slope of the linear relation between insulin (mU/L) and glucose (mg/dL) during the steady-state at each glucose clamp value (100 mg/dL, 150 mg/dL, and 200 mg/dL) [18]. β-Cell function was also measured as the clamp insulinogenic index: the ratio of the over-basal AUC_INSULIN_ to over-basal AUC_GLUCOSE_.

#### Resting metabolic rate

Resting metabolic rate was assessed as previously described [12] using indirect calorimetry. Data were analyzed using TurboFit (VacuMed, Ventura, CA).

#### Islet static incubation

Islets were isolated from the pancreas tail immediately before euthanasia [18, 19]. All *in vitro* experiments were performed after 18–24 h culture in 5-cm petri dishes. Prior to static incubation experiments, islets were washed three times in Krebs-Ringer bicarbonate buffer modified with HEPES [19, 20]. Islets were hand-picked and transferred to 24-well culture plates containing Krebs-Ringer buffer. After 1-h equilibrium period at 37°C with 3 mmol/L glucose, separate batches of islets (15 islets per well, three-replicate experiments) were exposed to either 3, 7, 10, 15, 20, or 27 mmol/L glucose for one additional hour (glucose solution was added to reach such concentrations). β-Cell function was calculated as 1) the total insulin secreted at the end of the static incubation (pmol•L^−1^/islet/h) and 2) the stimulation index: the ratio of the insulin secreted at high glucose concentration (second hour of the static incubation) to the basal insulin (equilibrium period) [19].

#### Liver gene expression

RNA was extracted from biopsied hepatic tissues using the Tri-Reagent Kit (Molecular Research Center, Cincinnati, OH). First-strand cDNA was synthesized according to manufacturer’s protocol, using 1 μg of total RNA using Superscript II (Invitrogen, Carlsbad, CA). Real-time polymerase-chain reaction (RT-PCR) was performed using a Light-Cycler 4.8 instrument (Roche Applied Science, Indianapolis, IN). cDNA was amplified using the ‘universal probe system’ on a Roche microplate with a final volume of 10 μL reaction mix containing 2.5 μL, 100-fold diluted cDNA, 7 μL LightCycler TaqMan Master Mix buffer (Roche Probes Master kit, Roche Applied Science, Indianapolis, IN), 1 μmol/L specific forward-reverse primers and 0.5 μL specific universal probes. Primers and universal probes are shown in Table 1. Quantification of 18S rRNA was used for sample normalization using TaqMan probes. RT-PCR was performed according to manufacturer’s protocol (Roche Applied science, Indianapolis, USA). The specificity of amplification was determined by melting curve analysis. The results were analyzed by relative quantification, ΔΔC method.

**Table 1:** Primers and probes used for RT-PCR.

Gene	Forward	Reverse	UPL
FAS	ATGCTGGGCATGGAGTTC	CACCAGTCCCATCACACG	59
SREBP1c	TGCTTCTGACAACCATGAAAA	GGCCAGGGAGCTGATACC	8
CPT1	ATGGGCATGAACGCAGAG	CAGGACGTACTCCCACAGGT	26
PPAR-α	GGAGCTAGATGACAGCGACA	GCGATCTCCACAGCAAATG	5
CEACAM1	TTCCAGAACATCACCCTGAA	AGTGCAGTTTCAAATTTTTGGTT	47
GK	GTGGCTGGAAAAGTTCAGGA	CACTCAGCACCACCAGTCC	161
GLUT2	AGCATCTTCGAACCTTGTCAC	TCATTCCACCAATTGCAAAG	93
GLUT4	CCCTATGTCTTCCTTCTGTTCG	CGGGTTTCAGGCACTTTTAG	15
G6Pase	AAGCCAATGACTGTGCCAAT	ACCTCTGGCCTCAAAATGG	83
PEPCK	GCTCCGAGGAGGAGAACC	CCTCTGATCATGCCCTGTC	67
18S rRNA	GCGGCTTTGGTGACTCTAGATA	TTGATAGGGCAGACGTTCG	141

18S rRNA, 18S ribosomal RNA; CEACAM1, carcinoembryonic antigen-related cell adhesion molecule 1; CPT1, carnitine palmitoyltransferase I; FAS, Fas cell surface death receptor; G6Pase, glucose-6-phosphatase; GK, glucokinase; GLUT2, glucose transporter type 2; GLUT4, glucose transporter type 4; PEPCK, phosphoenolpyruvate carboxykinase; PPAR-α, peroxisome proliferator-activated receptor alpha; RT-PCR, real-time polymerase-chain reaction; SREBP1c, sterol regulatory element-binding protein 1c; UPL, Universal Probe Library.

#### Blood sampling and biochemical assays

Blood samples were collected, stored, and analyzed as previously described [13, 18]. Insulin from plasma and *in vitro* experiments were determined by ELISA (human kit, Millipore) [19]. Glucagon concentration was measured by radioimmunoassay (canine kit, Millipore).

### Statistical analyses

Since data were non-normally distributed, non-parametric tests were used. Data were expressed as medians and their interquartile range (IQR). Wilcoxon matched-pairs signed-rank test and Mann-Whitney U test were used for comparison within and between groups, respectively. Quade’s test was used to compare the metabolic effects of exenatide versus placebo adjusting for pretreatment values. Mixed-effects linear regression [21]) was used to compare groups when repeated measures were performed, testing for treatment X time interaction. Friedman ANOVA was used if interaction was found to be significant. Differences were statistically significant if P was less than 0.05. All analyses were performed using IBM SPSS Statistics version 20.0 and Stata/SE 10.0 for Windows (StataCorp LP, College Station, TX).

## Results

### Pre-diabetes induction

STZ treatment resulted in a decrease in glucose tolerance (Table 2). Glycemia at 120 min post-oral glucose challenge increased by 22.0% (IQR, 31.5%) (P = 0.011). *K*_*G*_ decreased by 48.8% (36.9%) (P = 0.002). Fasting glycemia increased by 3.4% (IQR, 4.9%); P = 0.039), from 99.1 mg/dL (6.6 mg/dL) to 103.7 mg/dL (7.4 mg/dL). Fasting plasma insulin remained unchanged (P = 0.972). OGTT insulinogenic index decreased by 73.6% (IQR, 41.3%) (P = 0.011) and *AIR*_*G*_ fell by 54.0% (IQR, 40.7%) (P = 0.010). Since insulin sensitivity remained unchanged (P = 0.875), *DI* decreased accordingly (P = 0.006). *S*_*G*_ also decreased (P = 0.008). Body weight increased by 5.8% (IQR, 6.9%) (P = 0.046).

**Table 2:** Changes in metabolic parameters after induction of pre-diabetes following a single dose of streptozotocin (STZ) in high-fat-fed canines (n = 13).

	Before pre-diabetes	Pre-diabetes	Delta change	P value
***OGTT***				
Glucose 60 min (mg•dL^-1^)	110.7 (17.8)	148.0 (62.2)	43.1 (71.9)	0.002
Glucose 120 min (mg•dL^-1^)	97.0 (4.4)	123.1 (56.6)	18.6 (30.2)	0.011
AUC_GLUCOSE_ (mg•dL^-1^•min^-1^)	1972.0 (1589.0)	5337.0 (4817.0)	3365.0 (6071.0)	0.002
AUC_INSULIN_ (mU•L^-1^•min^-1^)	1707.0 (1402.0)	2040.0 (1269.0)	−154.0 (518.3)	0.422
Insulinogenic index (mU•L^-1^•min^-1^)/(mg•dl^-1^•min^-1^)	0.84 (0.67)	0.25 (0.31)	−0.58 (0.94)	0.011
***IVGTT***				
Fasting glucose (mg•dL^-1^)	99.1 (6.6)	103.7 (7.4)	−3.4 (5.1)	0.039
Fasting insulin (mU•L^-1^)	42.0 (30.0)	53.5 (32.3)	−1.3 (27.7)	0.972
*K*_*G*_ (%•min^-1^)*	3.3 (1.2)	1.6 (0.3)	−1.7 (1.6)	0.002
*AIR*_*G*_ (mU•L^-1^•min)*	481.9 (282.0)	178.8 (124.2)	−262.4 (200.8)	0.010
*S*_*I*_ (mU^-1^•L^-1^•min^-1^)*	4.7 (3.7)	4.7 (2.6)	−0.7 (5.1)	0.875
DI*	2476.4 (1586.1)	978.2 (520.2)	−1356.8 (1565.1)	0.006
*S*_*G*_ (%•min^-1^)*	4.2 (2.7)	2.7 (0.6)	−1.7 (2.3)	0.008
Body weight (kg)	27.1 (1.9)	27.8 (1.4)	1.5 (1.8)	0.046

Values are medians (interquartile range). Delta change column represents changes from baseline (before pre-diabetes).

*Due to partial missing insulin data, only 12 dogs were included for analyses.

P values were determined by Wilcoxon matched-pairs signed-rank test. *AIR*_*G*_, acute insulin response to glucose; AUC, area under the curve; *DI*, disposition index; IVGTT, intravenous glucose tolerance test; *K*_*G*_, intravenous glucose tolerance; OGTT, oral glucose tolerance test; *S*_*I*_, insulin sensitivity; *S*_*G*_, glucose effectiveness.

### Effect of exenatide on food intake and body composition

**Fig 1:**
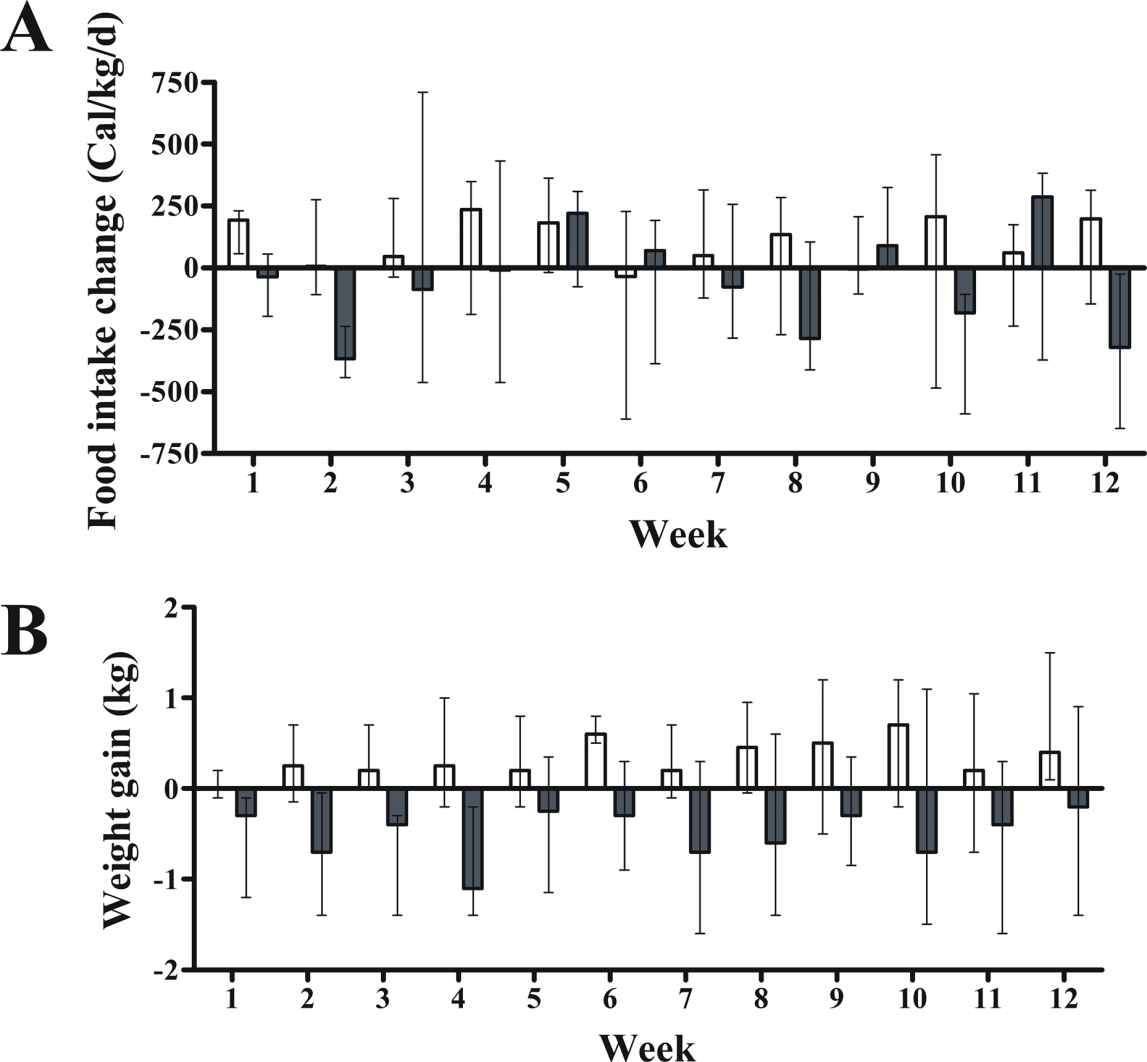
Effect of exenatide on food intake and body weight in pre-diabetic canines (n = 14). Overall, compared with placebo, 12-week treatment with exenatide had no effect on food intake (A) but did reduce body weight (B) throughout the study (P<0.001; mixed model regression). Gray and white columns indicate exenatide and placebo groups, respectively. Top of columns are medians. Bars are upper and lower quartiles.

Exenatide’s effect on food intake was not different from placebo throughout the study (P = 0.494; mixed model regression) (P = 0.603, Quade’s test) or at week 12 (Fig 1 and Table 3). In fact, there was a high variability in food intake (Fig 1A). In contrast, exenatide, compared with placebo, did promote mild body weight loss throughout the study (P<0.001; mixed model regression) (Fig 1B) with no effect on abdominal fat distribution (Table 3).

**Table 3:** Changes in food intake and body composition after exenatide treatment for 12 weeks in pre-diabetic canines.

	Exenatide (EXE) (n = 7)			Placebo (PBO) (n = 7)			P value (EXE vs. PBO)
	Baseline	Endpoint	Delta change	Baseline	Endpoint	Delta change	
*Food intake (Cal/d)*	2679.9 (1241.0)	2320.5 (579.6)	−322.2 (622.8)*	2320.8 (973.9)	2246.7 (1031.8)	−198.4 (459.0)	0.603
*Food intake (Cal/kg/d)*	98.5 (30.0)	84.4 (16.0)	−4.0 (18.7)*	84.7 (33.3)	79.8 (40.4)	6.4 (17.1)	0.624
*Body weight (kg)*	27.7 (3.5)	27.5 (3.1)	−0.2 (2.3)	27.9 (1.5)	28.8 (2.1)	0.4 (1.4)	0.217
*SAT (cm*^*3*^*)*	166.1 (63.1)	135.3 (73.4)	−2.1 (45.3)	227.2 (131.0)	191.3 (59.7)	−7.5 (56.4)	0.395
*VAT (cm*^*3*^*)*	310.2 (208.4)	312.5 (192.3)	−10.2 (68.8)	499.0 (194.4)	400.3 (316.8)	−0.8 (40.2)	0.445
*Total fat (cm*^*3*^*)*	504.5 (224.9)	492.5 (286.9)	−21.1 (72.5)	736.3 (185.7)	588.8 (371.5)	−6.3 (17.6)	0.325

Values are medians (interquartile range). Delta change columns represent changes from baseline in each group. VAT: visceral adipose tissue; SAT: subcutaneous adipose tissue.

* P<0.05, Wilcoxon matched-pairs signed-rank test. P values between groups were determined by Quade’s test, adjusting for baseline differences.

### Effect of exenatide on fasting plasma glucose, insulin, glucagon, and glycated hemoglobin A1c

**Fig 2:**
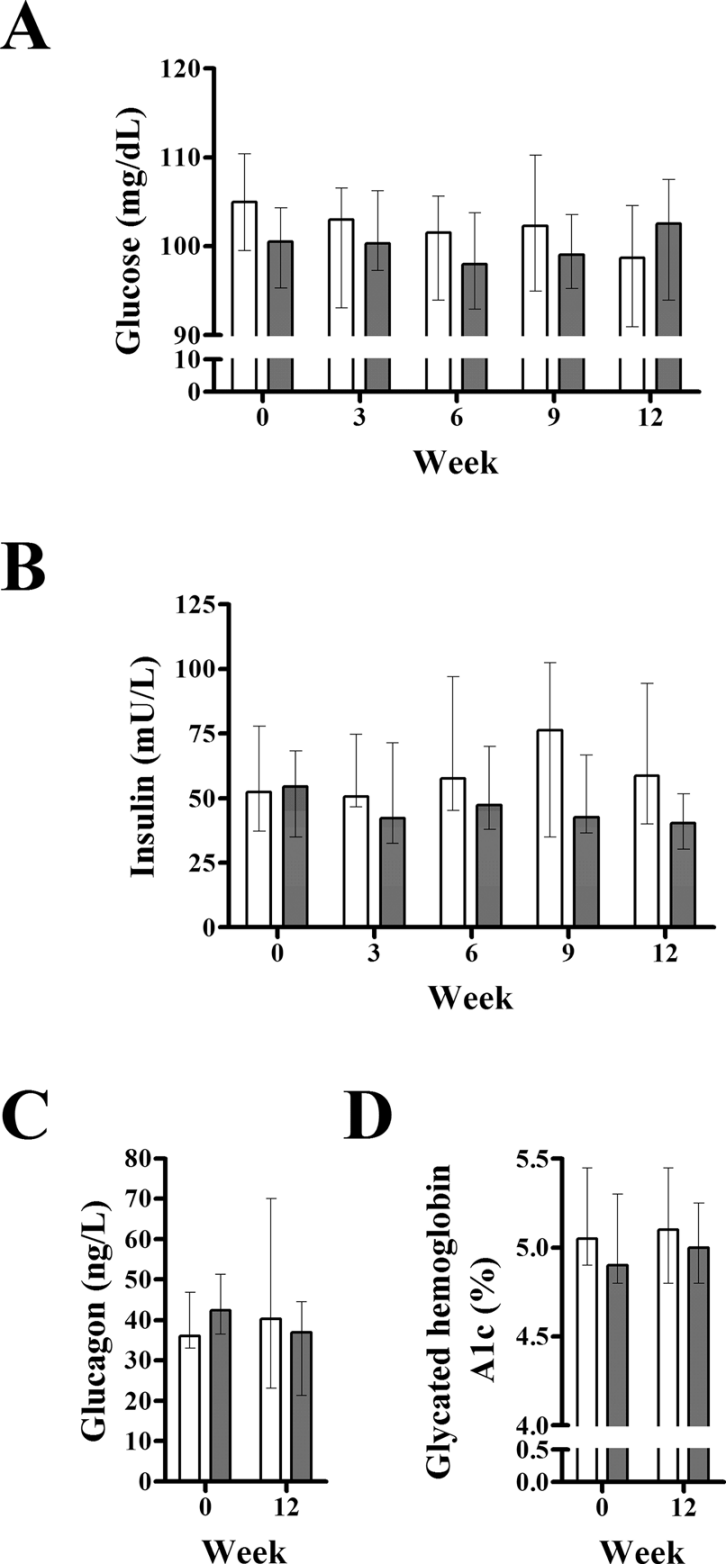
Effect of exenatide on fasting plasma glucose, insulin, glucagon, and glycated hemoglobin A1c. Compared to placebo (n = 7), exenatide (n = 7) did not alter fasting plasma glucose (A), insulin (B), glucagon (C) or glycated hemoglobin A1c (D) in pre-diabetic canines. Gray and white columns indicate exenatide and placebo groups, respectively. Top of columns are medians. Bars are upper and lower quartiles. In the placebo group, glucagon and hemoglobin A1c data were available only in 6 dogs.

Analysis of repeated measures showed no differences between groups in fasting plasma glucose (P = 0.663; mixed model) or insulin (P = 0.349) (Fig 2). However, since time interacted with treatment for insulin (P = 0.034) outcomes, Friedman ANOVA was performed independently. We found no changes in plasma insulin with placebo (P = 0.725) or exenatide (P = 0.150). Likewise, we found no changes in glycated hemoglobin A1c in the exenatide or placebo group (Fig 2).

### Effects of exenatide on glucose metabolism, insulin clearance, and energy expenditure

**Fig 3:**
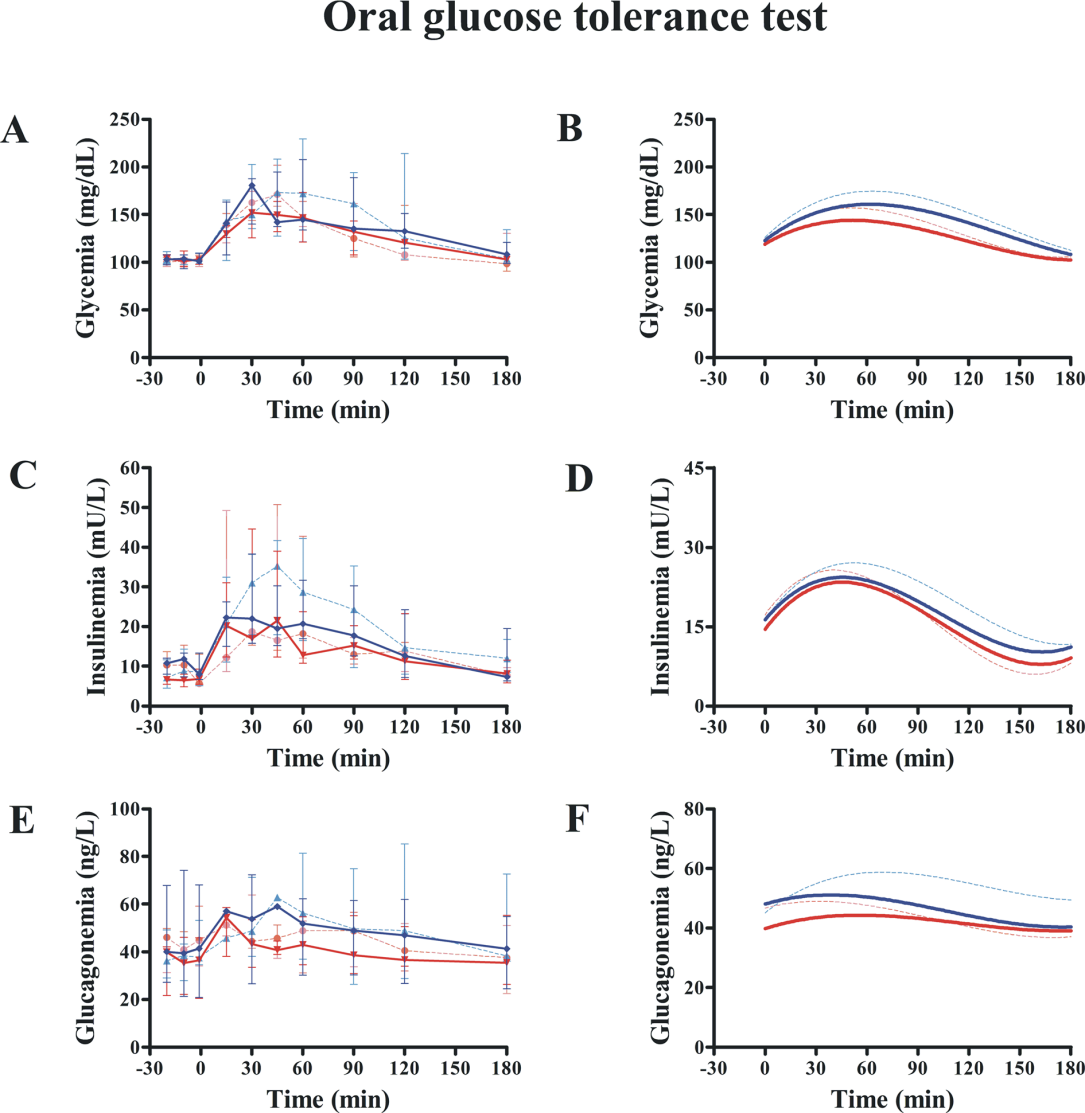
Effect of exenatide on oral glucose tolerance. Compared with placebo (n = 7), exenatide (n = 7) was not different in modifying AUC_GLUCOSE_ (A and B), AUC_INSULIN_ (C and D) or AUC_GLUCAGON_ (E and F). Continuous red and blue lines indicate exenatide and placebo groups, respectively, after 12 weeks. Dash red and blue lines indicate exenatide and placebo groups, respectively, at baseline (week 0). B, D, and F show the nonlinear fits (polynomial of third order) for curves represented in A, C, and E, respectively. Plots are medians. Bars are upper and lower quartiles. Glucagon data were available only in 6 dogs.

Exenatide treatment had no effect on oral glucose tolerance (Fig 3 and Table 4). Glycemia at 120 min post-oral glucose challenge remained unchanged (P = 1.00). Exenatide was not better than placebo in improving AUC_GLUCOSE_ (P = 0.063; Fig 3A and Fig 3B), AUC_INSULIN_ (P = 0.133; Fig 3C and Fig 3D) or AUC_GLUCAGON_ (P = 0.618; Fig 3E and Fig 3F). Likewise, *K*_*G*_ was not different between groups (P = 0.658, mixed model). However, compared with placebo, exenatide had a beneficial effect on OGTT insulinogenic index (P = 0.040, Quade’s test) (Table 4), even while adjusting for body weight (P = 0.032, mixed model).

**Table 4:** Changes in metabolic parameters after exenatide treatment for 12 weeks in pre-diabetic canines.

	Exenatide (EXE) (n = 7)			Placebo (PBO) (n = 7)			P value (EXE vs. PBO
	Baseline	Endpoint	Delta change	Baseline	Endpoint	Delta change	
***OGTT***							
Glucose 60 min (mg•dL^-1^)	147.0 (26.9)	146.6 (52.2)	−17.3 (35.2)	172.2 (107.7)	144.9 (73.7)	−6.6 (42.1)	0.956
Glucose 120 min (mg•dL^-1^)	107.5 (57.8)	120.7 (5.0)	13.6 (63.4)	125.4 (110.8)	132.5 (36.7)	−11.3 (74.7)	0.298
AUC_GLUCOSE_ (mg•dL^-1^•min^-1^)	5337.0 (4225.0)	4367.0 (3623.0)	−1815.0 (5068.0)	6617.0 (11311.0)	7523.0 (6729.0)	92.(6186.0)	0.063
AUC_INSULIN_ (mU•L^-1^•min^-1^)	1139.0 (2047.3)	1517.0 (1994.0)	−39.8 (994.3)	2075.0 (980.0)	1377.0 (1507.3)	−576.9 (1540.4)	0.133
AUC_GLUCAGON_ (ng•L^-1^•min^-1^)	0.17 (0.20)	0.39 (1.41)	0.36 (0.96)	0.25 (0.35)*	0.44 (0.37)*	0.02 (0.70)	0.618
Insulinogenic index (mU•L^-1^•min^-1^)/(mg•dl^-1^•min^-1^)	0.20 (0.51)	0.40 (0.74)	0.18 (0.32)	0.29 (0.31)	0.18 (0.11)	−0.05 (0.18)	0.040
**IVGTT**							
*K*_*G*_ (%•min^-1^)	1.5 (0.5)	1.5 (0.9)	0.3 (0.8)	1.6 (0.4)	2.5 (1.7)	0.9 (2.0)	0.377
*AIR*_*G*_ (mU•L^-1^•min)	158.7 (280.3)	278.4 (200.7)	−25.0 (175.2)	183.5 (104.4)	209.9 (154.7)	24.9 (87.4)	0.266
*S*_*I*_ (mU^-1^•L^-1^•min^-1^)	4.3 (2.4)	5.2 (2.7)	0.7 (2.4)	5.2 (3.4)	3.3 (5.0)	−0.5 (4.2)	0.274
*DI*	1058.7 (574.3)	1094.7 (597.7)	106.2 (441.4)	946.7 (1002.8)	840.8 (858.6)	77.2 (398.6)	0.521
*S*_*G*_ (%•min^-1^)	2.4 (0.8)	3.0 (1.1)	0.5 (0.4)†	2.9 (0.8)	3.3 (1.6)	0.2 (1.4)	0.297
*MCR* (mL•min^-1^•kg^-1^)	8.5 (5.4)	11.8 (3.1)	0.5 (4.9)	10.1 (2.4)	8.7 (1.7)	−1.4 (3.5)	0.116
*FCR* (min^-1^)	0.47 (0.20)	0.40 (0.07)	−0.09 (0.20)	0.47 (0.10)	0.42 (0.24)	−0.15 (0.31)	0.851
**Graded-Hyperglycemic clamp**							
Insulin/Glucose slope (mU•L^-1^)/(mg•dl^-1^)	20.5 (29.5)	25.5 (17.3)	−6.9 (21.7)	22.5 (13.9)	23.1 (13.9)	0.6 (8.7)	0.498
AUC_GLUCOSE_ (baseline-200 mg•dL^-1^)	11894.0 (5430.0)	11592.0 (2312.0)	−365.0 (3075.0)	11716.0 (1988.0)	10450.0 (1656.0)	−1019.0 (2970.0)	0.335
AUC_INSULIN_ (baseline-200 mg•dL^-1^)	2296.0 (1949.0)	2663.0 (2150.0)	−73.0 (849.0)	2407.0 (2352.0)	1839.0 (2059.0)	27.0 (1981.0)	0.586
Insulinogenic index (baseline-200 mg•dL^-1^)	0.16 (0.29)	0.22 (0.28)	−0.00 (0.19)	0.21 (0.28)	0.18 (0.22)	0.02 (0.20)	0.534
AUC_GLUCOSE_ (100–150 mg•dL^-1^)	1143.0 (327.5)	822.8 (428.4)	−177.6 (755.9)	854.1 (387.6)	1103.0 (601.4)	72.0 (531.3)	0.381
AUC_INSULIN_ (100–150 mg•dL^-1^)	365.1 (178.5)	294.3 (505.8)	−28.2 (317.1)	272.8 (672.6)	212.3 (261.5)	−56.9 (184.1)	0.567
Insulinogenic index (100–150 mg•dL^-1^)	0.30 (0.15)	0.41 (0.48)	0.11 (0.25)†	0.32 (0.61)	0.19 (0.19)	−0.05 (0.19)	0.016
**Energy expenditure**							
Resting metabolic rate (Cal/kg/d)	42.2 (9.0)	40.0 (16.3)	−3.9 (9.7)	49.0 (13.2)	48.2 (7.6)	−1.0 (13.5)	0.667
Energy from carbohydrate oxidation (%)	25.1 (29.8)	43.2 (14.9)	17.0 (27.1)†	38.6 (37.8)	45.5 (41.1)	9.0 (62.7)	0.867

Values are medians (interquartile range). Delta change columns represent changes from baseline in each group.

* Data available only for 6 dogs.

† P<0.05, Wilcoxon matched-pairs signed-rank test.

*AIR*_*G*_, acute insulin response to glucose; AUC, area under the curve; *DI*, disposition index; *FCR*, fractional clearance rate of insulin; IVGTT, intravenous glucose tolerance test; *K*_*G*_, intravenous glucose tolerance; *MCR*, metabolic clearance rate of insulin; OGTT, oral glucose tolerance test; *S*_*I*_, insulin sensitivity; *S*_*G*_, glucose effectiveness.

**Fig 4:**
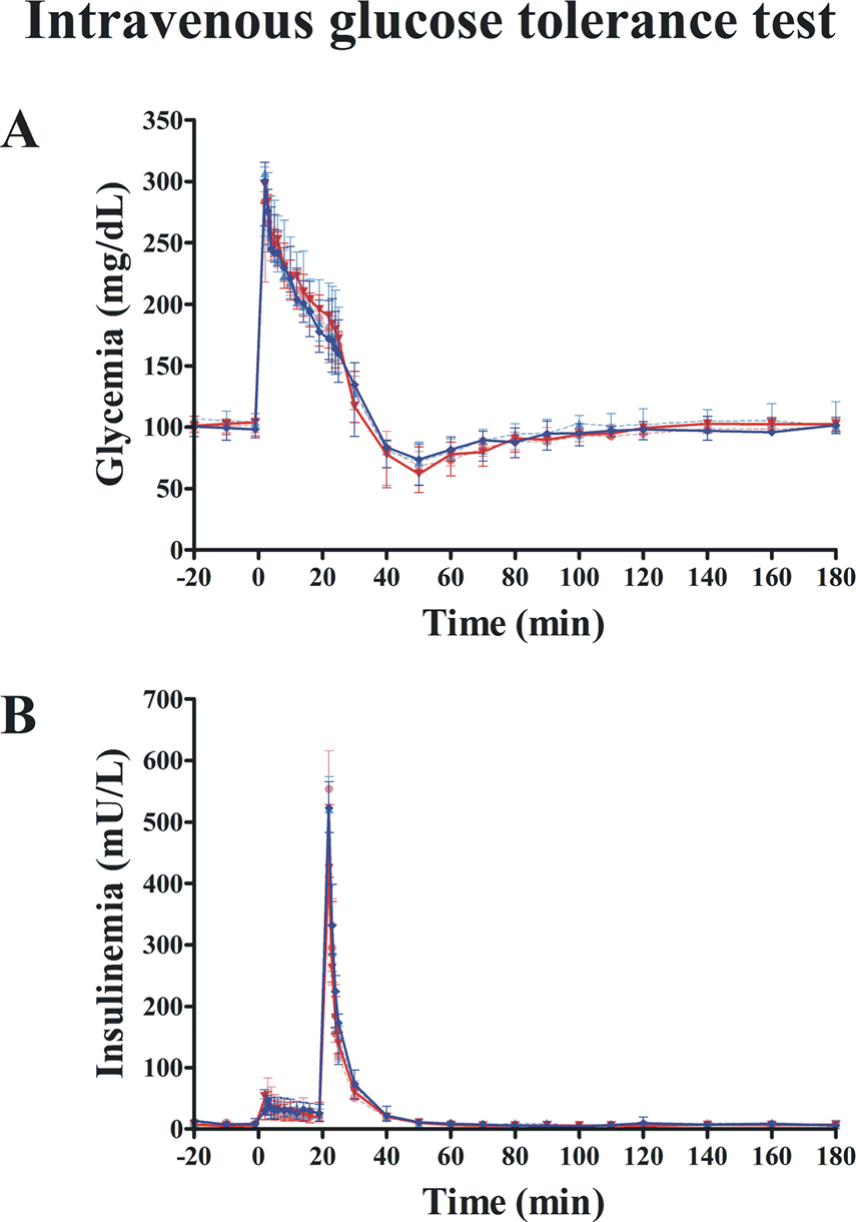
Effect of exenatide on intravenous glucose tolerance. Glucose (A) and insulin (B) profiles are identical in exenatide (n = 7) and placebo groups (n = 7). Continuous red and blue lines indicate exenatide and placebo groups, respectively, after 12 weeks. Dash red and blue lines indicate exenatide and placebo groups, respectively, at baseline (week 0).

**Fig 5:**
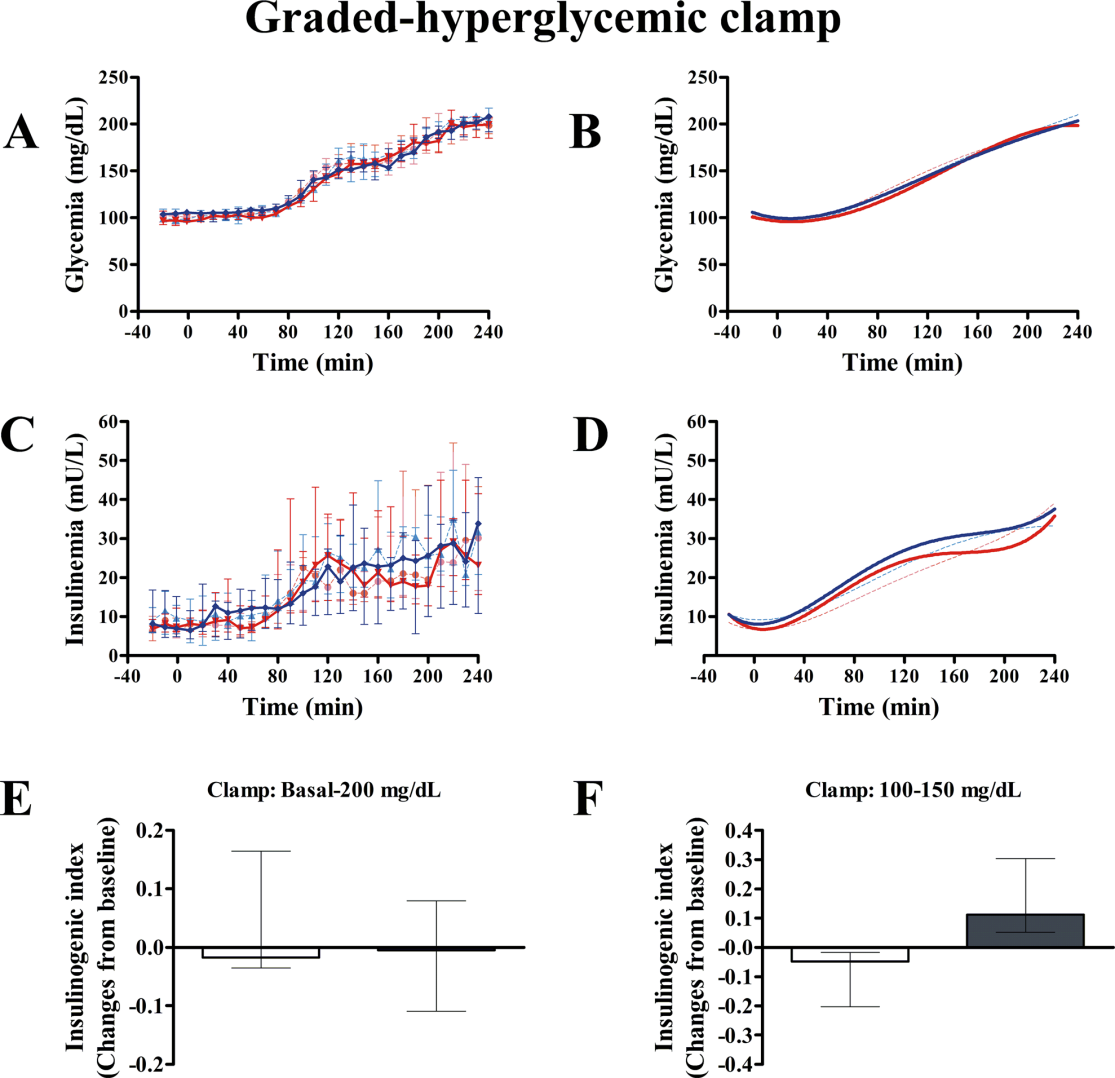
Effect of exenatide on β-cell function during graded-hyperglycemic clamp. After 12 weeks, compared with placebo (n = 7), exenatide (n = 7) was not different in modifying the area under the curve of glucose (AUC_GLUCOSE_) (A and B) or AUC_INSULIN_ (C and D) during the whole test. In E and F, β-cell function is expressed as the insulinogenic index: the ratio of AUC_INSULIN_ to AUC_GLUCOSE_. Clamp at very high glucose concentrations does not discriminate the stimulatory effect of exenatide on β-cell function, as compared with placebo (E). In contrast, clamp period at more physiological glucose concentrations (F) shows a significant increase in β-cell function as compared with placebo (P = 0.016, Quade’s test). In A-D, continuous red and blue lines indicate exenatide and placebo groups, respectively. Dash red and blue lines indicate exenatide and placebo groups, respectively, at baseline (week 0). B and D show the nonlinear fits (polynomial of third order) for curves represented in A and C, respectively. In E and F, gray and white columns indicate exenatide and placebo groups, respectively. Columns are medians. Bars are upper and lower quartiles.

Data calculated from the IVGTT (Fig 4) showed no exenatide effects on *AIR*_*G*_, *S*_*I*_, *DI* or *S*_*G*_ when compared with placebo (Table 4). Likewise, we found no differences between groups in insulin clearance throughout the study (*MCR*: P = 0.952; *FCR*: P = 0.499; mixed model). The insulin/glucose slope calculated from the graded-hyperglycemic clamp was not different between groups (P = 0.498) (Table 4). Likewise, clamp insulinogenic index from t = -20 to t = 240 was not different between groups (P = 0.534) (Fig 5). However, we noted that the clamp insulinogenic index estimated from t = 40 to t = 110 (period when the median plasma glucose actually increased from ~100 mg/dL to ~150 mg/dL), improved with exenatide (P = 0.043 vs. baseline; P = 0.016, between groups, Quade’s test) (Fig 5 and Table 4). We did not see changes in β-cell function from t = −20 to t = 30 (P = 0.492, Quade’s test). Likewise, we did not see changes in β-cell function from t = 120 to t = 240 (when the median glucose increased from ~150 mg/dL to 200 mg/dL) (P = 0.753, Quade’s test).

Although we found no differences in resting metabolic rate (Table 4), exenatide group showed an increase in carbohydrate oxidation (P = 0.046). However, this effect was not different compared with placebo (P = 0.867, Quade’s test).

### Effect of exenatide on β-cell and liver function *in vitro*

**Fig 6:**
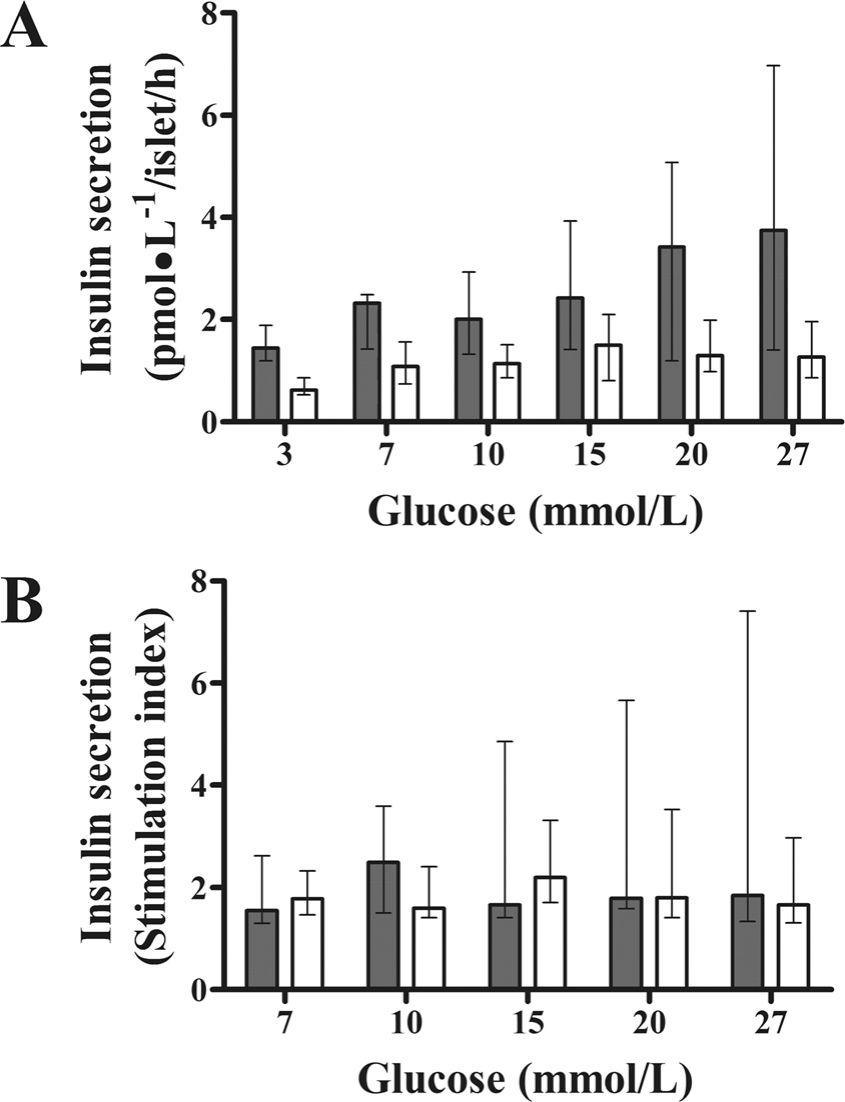
Effect of chronic systemic treatment with exenatide on β-cell function assessed *in vitro*. Compared with placebo, 12-week systemic treatment with exenatide improved total insulin secretion in isolated pancreatic islets from pre-diabetic canines (A) (P = 0.041, mixed model). Each glucose concentration was tested in different islet batches (exenatide: n = 5; placebo: n = 4; three-replicate experiments) (A). Note that the differences in insulin secretion between groups disappeared when insulin secretion was normalized to basal insulin (P = 0.523, mixed model) (B). Gray and white columns indicate exenatide and placebo groups, respectively. Top of columns are medians. Bars are upper and lower quartiles.

Exenatide, compared with placebo, improved glucose-stimulated insulin secretion *in vitro* (P = 0.041, mixed model) (Fig 6), likely influenced by increased basal insulin in the exenatide group (P = 0.046) (Fig 6A). When glucose-stimulated insulin secretion was adjusted for basal insulin, the differences between groups disappeared (P = 0.184). β-Cell function expressed as the stimulation index was not different between groups (P = 0.523; Fig 6B).

Since an increase in carbohydrate oxidation occurred with exenatide, we measured mRNA expression of liver genes related to fat and glucose metabolism. We found no significant differences between groups in genes related to fatty acid synthesis (Fas cell surface death receptor: P = 0.291; sterol regulatory element-binding protein 1c: P = 0.685), glucose oxidation (glucose transporter type 2: P = 0.223; glucose transporter type 4: P = 0.391; glucokinase: P = 0.062), fat oxidation (carnitine palmitoyltransferase I: P = 0.123; peroxisome proliferator-activated receptor alpha: P = 0.062); or insulin clearance (carcinoembryonic antigen-related cell adhesion molecule 1: P = 0.223). Although we found lower mRNA expression of glucose-6-phosphatase (P = 0.028) in the exenatide group, as compared with placebo, mRNA expression of phosphoenolpyruvate carboxykinase was not significantly different (P = 0.116); thus, our data do not conclusively support an inhibitory effect of exenatide on gluconeogenic activity.

## Discussion

Our findings indicate that a 12-week treatment with exenatide alone, compared with placebo, improves β-cell function but not glucose tolerance or insulin sensitivity in pre-diabetic canines fed a HFD. Improvement of β-cell function did not translate in lower fasting glycemia or reduced glycated hemoglobin A1c concentration. These results indicate only partial beneficial metabolic effects of exenatide alone on pre-diabetes.

Numerous studies have explored the therapeutical effect of exenatide in type 2 diabetes [3]. However, a few studies have explored the effect of exenatide on glucose homeostasis on pre-diabetes. Exenatide has been shown to improve glucose homeostasis in individuals with pre-diabetes in combination with lifestyle changes or other antidiabetic drugs [8–10]. However, whether the beneficial effects of chronic exenatide treatment on pre-diabetes are mainly due to exenatide *per se* have remained unclear. In fact, no previous study has specifically explored the effect of exenatide alone on glucose tolerance, β-cell function, and insulin sensitivity in the pre-diabetes state, using well accepted methods, under controlled conditions. In the present study, we used a canine model of pre-diabetes, characterized by impaired glucose tolerance as evidenced by a ~20% increase in plasma glucose at minute 120 during OGTT and a reduction in *K*_*G*_ by ~50%. In addition, our pre-diabetic canines showed mild impaired fasting glucose, as evidenced by a ~3% increase in fasting glycemia.

Chronic exenatide treatment, compared with placebo, caused a reduction in body weight while animals where maintained on a HFD. These results are consistent with previous findings in individuals with impaired fasting glucose or impaired glucose tolerance [8–10]. However, we found no beneficial effect of exenatide on abdominal fat mass. Exenatide alone given for 12 weeks has been shown to promote weight loss in obese individuals by reducing appetite rather than increasing energy expenditure [22]. We did not find changes in food intake or resting metabolic rate in pre-diabetic canines. Thus, the mechanisms by which chronic treatment with exenatide induced weight loss in the pre-diabetic canines remain elusive, though physical activity or energy expenditure were not measured, also plausible mechanisms for weight loss. Nevertheless, exenatide alone appears to cause a very modest body weight loss in individuals with pre-diabetes [10], and in individuals with severe obesity [22], which is consistent with our findings (Fig 1B). Exenatide combined with diet and physical exercise may well have a stronger effect in reducing body weight in individuals with pre-diabetes [8].

We did not find changes in plasma fasting glucose or glycated hemoglobin A1c, which is consistent with findings from large clinical studies with exenatide in pre-diabetic individuals [8, 10]. In individuals with type 2 diabetes, a significant but small decrease in glycated hemoglobin A1c has been reported after exenatide treatment concomitant with metformin (−0.4%) for 102–113 weeks [23] or with sulfonylurea (−0.8%) over a period of 30 weeks [24].

We found an improvement of β-cell function during OGTT (independent of body weight) but not during the IVGTT after exenatide treatment. These differences could be related, at least in part, to exenatide’s incretin effect [25]. This finding is consistent with those from clinical studies conducted in pre-diabetic [9] and diabetic individuals [26]. However, we also found an improvement of β-cell function assessed by the hyperglycemic clamp at more moderated intravenous glucose incursions (up to 150 mg/dL). This might suggest an incretin-independent effect of exenatide on the β-cells or reduced insulin clearance. However, the latter possibility is unlikely since we did not detect changes in *MCR* or *FCR*.

It is surprising that improvement of β-cell function by exenatide did not translate in an improvement in glucose tolerance. Previous studies have shown improved glucose tolerance with exenatide in pre-diabetic individuals [8, 9]; however, it is not clear in the latter studies if the beneficial effect on glucose tolerance was due to exenatide *per se* or due to the effect of lifestyle intervention (diet and exercise) [8] or the combination with pioglitazone and metformin [9]. It is possible that improvements in insulin sensitivity are necessary to observe substantial improvement in glucose tolerance. However, insulin resistance and β-cell dysfunction, independently, appears to be predictive of glucose intolerance in humans [27].

We did not observe changes in insulin sensitivity, arguing the findings from a previous clinical study in pre-diabetic patients [9]; however, in the latter study exenatide was given in combination with pioglitazone and metformin, the former drug known to be an insulin sensitizer [28]. Individuals with new onset type 2 diabetes receiving exenatide for 24 weeks improved insulin sensitivity, while following a diet and exercise regimen [29]. Positive effect in canines [30] or no effect in humans [31] on insulin sensitivity have been reported after acute administration of exenatide. Rodent studies have shown a beneficial effect of exenatide on insulin sensitivity at very high concentrations after 6–10 weeks [32, 33]. *In vitro* studies also show contradictory results [34, 35]. It is unclear whether the conflicting results are due to differences in exenatide dose, duration of treatment, health conditions of study participants or species-related differences. Thus, the possible beneficial effect of exenatide on insulin sensitivity remains controversial. It should be noted that HFD did not decrease insulin sensitivity in our pre-diabetic canines, possibly explained by a reduced anabolic effect of insulin post-STZ, potentially preventing further improvement of insulin sensitivity by exenatide.

Exendin-4 has a direct insulinotropic effect in rat islets [36, 37], mouse insulinoma cells [37], and islets from type 2 diabetic subjects [38]. Isolated human islets cultured in presence of exenatide for 48 h also showed increased insulin secretion [39, 40]. However, the effect of chronically administered exenatide on isolated islets has not previously been explored in a model of pre-diabetes. Activation of the glucagon-like peptide 1 receptor may also promote β-cell proliferation and prevent β-cell apoptosis, particularly in rodents [41, 42], whereas those effects may not necessarily translate to humans [42, 43]. Since we did not assess β-cell mass, we cannot prove or disprove a possible contribution of increased β-cell mass to explain the increased in vitro insulin secretion in the present study. However, supporting a potential contribution of increased β-cell mass is the fact that we did not find differences in glucose-stimulated insulin secretion in vitro when data were normalized to basal insulin secretion. Since the in vitro insulinotropic effect of exenatide appears to be transient while exenatide is present in the media [40, 44], the increased insulin secretion in vitro in our study does not appear to be explained by an acute effect of exenatide since islets were tested 18–24 h after isolation in the absence of exenatide in vitro. Nevertheless, the argument in favor of increased β-cell mass after chronic exenatide treatment in canines is purely speculative.

The present study has strengths. No previous study has specifically explored the chronic effect of exenatide alone on glucose tolerance, β-cell function, and insulin sensitivity in an animal model of pre-diabetes. Chronic exenatide therapy in combination with lifestyle has been reported to have beneficial effects on pre-diabetes [8–10]. However, whether these effects are mainly due to exenatide *per se* or lifestyle changes have remained unclear. In the present study we demonstrated that chronic exenatide treatment alone improves β-cell function but not glucose tolerance or insulin sensitivity. Strength of the present study is that we used a relatively homogenous population of animals with a pre-diabetic state induced ~5 weeks prior to the commencement of the exenatide treatment. Previous studies [8–10] have explored exenatide’s effects on glucose homeostasis in individuals with pre-diabetes of variable duration.

Our findings should be interpreted according to the limitations of the study. Our experiments were performed in a small number of animals. Unlike the frequent association of insulin resistance with impaired glucose tolerance in humans [45], our canine model of pre-diabetes did not develop insulin resistance, even while animals were fed a HFD. In addition, our canine model of pre-diabetes was very mild, therefore, diagnostic criteria for human pre-diabetes [46] cannot be applied. However, although small, we induced a significant increase in fasting glycemia and glycemia 120 min post-OGTT (Table 2), and a marked decrease in intravenous glucose tolerance or *K*_*G*_ [47]. Another limitation is the relatively short duration of exenatide treatment (12 weeks). It is uncertain whether a more prolonged exenatide treatment in pre-diabetic canines would have resulted in normalization of glucose intolerance and fasting hyperglycemia.

Because we used STZ, it can be argued that our model of pre-diabetes may resemble a pre-diabetic model of type 1 diabetes, rather than a pre-diabetic model of type 2 diabetes. However, we administered a single low dose of STZ (18.5 mg/kg), much lower than the high total STZ dose used in rodents to induce type 1 diabetes (typically ranging from 150–200 mg/kg [48]), aiming to partially impair β-cell function and to induce glucose intolerance and mild hyperglycemia. Moreover, the animals studied were fed a HFD to resemble obesity, a well-established risk factor for type 2, but not type 1, diabetes. Thus, our data put in perspective the partial beneficial metabolic effects of chronic exenatide treatment alone, a widely used clinical drug, in a canine model of pre-diabetes. However, clinical studies are required to determine whether exenatide alone would be appropriate as a pre-diabetes therapy.

In conclusion, in our pre-diabetic canine model, 12-week exenatide treatment improved β-cell function but not glucose tolerance or insulin sensitivity. These findings demonstrate partial beneficial metabolic effects of exenatide alone on an animal model of pre-diabetes.

## References

[1] Bergman RN. Orchestration of glucose homeostasis: from a small acorn to the California oak. Diabetes. 2007;56: 1489–501. 17526912 10.2337/db07-9903

[2] Prasad-Reddy L, Isaacs D. A clinical review of GLP-1 receptor agonists: efficacy and safety in diabetes and beyond. Drugs Context. 2015;4: 212283 10.7573/dic.212283 26213556 PMC4509428

[3] Tella SH, Rendell MS. Glucagon-like polypeptide agonists in type 2 diabetes mellitus: efficacy and tolerability, a balance. Ther Adv Endocrinol Metab. 2015;6: 109–34. 10.1177/2042018815580257 26137215 PMC4480552

[4] Nathan DM, Davidson MB, DeFronzo RA, Heine RJ, Henry RR, Pratley R, et al Impaired fasting glucose and impaired glucose tolerance: implications for care. Diabetes Care. 2007;30: 753–9. 17327355 10.2337/dc07-9920

[5] ADA. 2. Classification and Diagnosis of Diabetes. Diabetes Care. 2015;38: S8–S16. 10.2337/dc15-S005 25537714

[6] Selph S, Dana T, Blazina I, Bougatsos C, Patel H, Chou R. Screening for type 2 diabetes mellitus: a systematic review for the U.S. Preventive Services Task Force. Ann Intern Med. 2015;162: 765–76. 10.7326/M14-2221 25867111

[7] Colagiuri S. Epidemiology of prediabetes. Med Clin North Am. 2011;95: 299–307, vii. 10.1016/j.mcna.2010.11.003 21281834

[8] Rosenstock J, Klaff LJ, Schwartz S, Northrup J, Holcombe JH, Wilhelm K, et al Effects of exenatide and lifestyle modification on body weight and glucose tolerance in obese subjects with and without pre-diabetes. Diabetes Care. 2010;33: 1173–5. 10.2337/dc09-1203 20332357 PMC2875418

[9] Armato J, DeFronzo RA, Abdul-Ghani M, Ruby R. Successful treatment of prediabetes in clinical practice: targeting insulin resistance and beta-cell dysfunction. Endocr Pract. 2012;18: 342–50. 10.4158/EP11194.OR 22068250

[10] Kelly AS, Bergenstal RM, Gonzalez-Campoy JM, Katz H, Bank AJ. Effects of exenatide vs. metformin on endothelial function in obese patients with pre-diabetes: a randomized trial. Cardiovasc Diabetol. 2012;11: 64 10.1186/1475-2840-11-64 22681705 PMC3434036

[11] Karve A, Hayward RA. Prevalence, diagnosis, and treatment of impaired fasting glucose and impaired glucose tolerance in nondiabetic U.S. adults. Diabetes Care. 2010;33: 2355–9. 10.2337/dc09-1957 20724649 PMC2963494

[12] Richey JM, Woolcott OO, Stefanovski D, Harrison LN, Zheng D, Lottati M, et al Rimonabant prevents additional accumulation of visceral and subcutaneous fat during high-fat feeding in dogs. Am J Physiol Endocrinol Metab. 2009;296: E1311–1318. 10.1152/ajpendo.90972.2008 19366874 PMC3833919

[13] Ionut V, Liu H, Mooradian V, Castro AV, Kabir M, Stefanovski D, et al Novel Canine Models of Obese Pre-Diabetes and of Mild Type 2 Diabetes. Am J Physiol Endocrinol Metab. 2010;298: E38–48. 10.1152/ajpendo.00466.2009 19843874 PMC2806110

[14] Ionut V, Castro AVB, Woolcott OO, Stefanovski D, Iyer MS, Broussard JL, et al Hepatic portal vein denervation impairs oral glucose tolerance but not exenatide's effect on glycemia. Am J Physiol Endocrinol Metab. 2014;307: E644–E652. 10.1152/ajpendo.00244.2014 25117408 PMC4200304

[15] Stefanovski D, Richey JM, Woolcott O, Lottati M, Zheng D, Harrison LN, et al Consistency of the Disposition Index in the Face of Diet Induced Insulin Resistance: Potential Role of FFA. PLoS One. 2011;6: e18134 10.1371/journal.pone.0018134 21479217 PMC3068147

[16] Castro AVB, Woolcott OO, Iyer MS, Kabir M, Ionut V, Stefanovski D, et al Increase in visceral fat per se does not induce insulin resistance in the canine model. Obesity. 2015;23: 105–11. 10.1002/oby.20906 25322680 PMC4276477

[17] Ader M, Stefanovski D, Kim SP, Richey JM, Ionut V, Catalano KJ, et al Hepatic insulin clearance is the primary determinant of insulin sensitivity in the normal dog. Obesity (Silver Spring). 2014;22: 1238–45. 24123967 10.1002/oby.20625 PMC3969862

[18] Woolcott OO, Richey JM, Kabir M, Chow RH, Iyer MS, Kirkman EL, et al High-fat diet-induced insulin resistance does not increase plasma anandamide levels or potentiate anandamide insulinotropic effect in isolated canine islets. PLoS One. 2015;10: e0123558 10.1371/journal.pone.0123558 25855974 PMC4391925

[19] Woolcott OO, Bergman RN, Richey JM, Kirkman EL, Harrison LN, Ionut V, et al Simplified method to isolate highly pure canine pancreatic islets. Pancreas. 2012;41: 31–38. 10.1097/MPA.0b013e318221fd0e 21792087 PMC4423806

[20] Woolcott OO, Gustafsson AJ, Dzabic M, Pierro C, Tedeschi P, Sandgren J, et al Arachidonic acid is a physiological activator of the ryanodine receptor in pancreatic beta-cells. Cell Calcium. 2006;39: 529–537. 16620964 10.1016/j.ceca.2006.02.003

[21] Hamilton LC. Multilevel and mixed-effects modeling. In: eds. Statistics with STATA: Updated for version 10 Belmont: Brooks/Cole; 2008 pp. 387–421.

[22] Bradley DP, Kulstad R, Racine N, Shenker Y, Meredith M, Schoeller DA. Alterations in energy balance following exenatide administration. Appl Physiol Nutr Metab. 2012;37: 893–9. 10.1139/h2012-068 22735035 PMC3623676

[23] Gallwitz B, Guzman J, Dotta F, Guerci B, Simo R, Basson BR, et al Exenatide twice daily versus glimepiride for prevention of glycaemic deterioration in patients with type 2 diabetes with metformin failure (EUREXA): an open-label, randomised controlled trial. Lancet. 2012;379: 2270–8. 10.1016/S0140-6736(12)60479-6 22683137

[24] Kendall DM, Riddle MC, Rosenstock J, Zhuang D, Kim DD, Fineman MS, et al Effects of exenatide (exendin-4) on glycemic control over 30 weeks in patients with type 2 diabetes treated with metformin and a sulfonylurea. Diabetes Care. 2005;28: 1083–91. 15855571 10.2337/diacare.28.5.1083

[25] Holst JJ. The physiology of glucagon-like peptide 1. Physiol Rev. 2007;87: 1409–39. 17928588 10.1152/physrev.00034.2006

[26] DeFronzo RA, Triplitt C, Qu Y, Lewis MS, Maggs D, Glass LC. Effects of exenatide plus rosiglitazone on beta-cell function and insulin sensitivity in subjects with type 2 diabetes on metformin. Diabetes Care. 2010;33: 951–7. 10.2337/dc09-1521 20107105 PMC2858197

[27] Weyer C, Tataranni PA, Bogardus C, Pratley RE. Insulin resistance and insulin secretory dysfunction are independent predictors of worsening of glucose tolerance during each stage of type 2 diabetes development. Diabetes Care. 2001;24: 89–94. 11194248 10.2337/diacare.24.1.89

[28] Waugh J, Keating GM, Plosker GL, Easthope S, Robinson DM. Pioglitazone: a review of its use in type 2 diabetes mellitus. Drugs. 2006;66: 85–109. 16398569 10.2165/00003495-200666010-00005

[29] Gastaldelli A, Brodows RG, D'Alessio D. The effect of chronic twice daily exenatide treatment on beta-cell function in new onset type 2 diabetes. Clin Endocrinol (Oxf). 2014;80: 545–53. 23574529 10.1111/cen.12199

[30] Zheng D, Ionut V, Mooradian V, Stefanovski D, Bergman RN. Exenatide sensitizes insulin-mediated whole-body glucose disposal and promotes uptake of exogenous glucose by the liver. Diabetes. 2009;58: 352–9. 10.2337/db08-0875 19011168 PMC2628608

[31] Vella A, Shah P, Reed AS, Adkins AS, Basu R, Rizza RA. Lack of effect of exendin-4 and glucagon-like peptide-1-(7,36)-amide on insulin action in non-diabetic humans. Diabetologia. 2002;45: 1410–5. 12378382 10.1007/s00125-002-0924-4

[32] Gedulin BR, Nikoulina SE, Smith PA, Gedulin G, Nielsen LL, Baron AD, et al Exenatide (exendin-4) improves insulin sensitivity and {beta}-cell mass in insulin-resistant obese fa/fa Zucker rats independent of glycemia and body weight. Endocrinology. 2005;146: 2069–76. 15618356 10.1210/en.2004-1349

[33] Lotfy M, Singh J, Rashed H, Tariq S, Zilahi E, Adeghate E. Mechanism of the beneficial and protective effects of exenatide in diabetic rats. J Endocrinol. 2014;220: 291–304. 10.1530/JOE-13-0426 24353307

[34] Wang L, Guo F, Wei S, Zhao R. Divergent effects of GLP-1 analogs exendin-4 and exendin-9 on the expression of myosin heavy chain isoforms in C2C12 myotubes. Peptides. 2011;32: 1313–9. 10.1016/j.peptides.2011.03.018 21453734

[35] Idris I, Patiag D, Gray S, Donnelly R. Exendin-4 increases insulin sensitivity via a PI-3-kinase-dependent mechanism: contrasting effects of GLP-1. Biochem Pharmacol. 2002;63: 993–6. 11911852 10.1016/s0006-2952(01)00924-8

[36] Parkes DG, Pittner R, Jodka C, Smith P, Young A. Insulinotropic actions of exendin-4 and glucagon-like peptide-1 in vivo and in vitro. Metabolism. 2001;50: 583–9. 11319721 10.1053/meta.2001.22519

[37] Goke R, Fehmann HC, Linn T, Schmidt H, Krause M, Eng J, et al Exendin-4 is a high potency agonist and truncated exendin-(9–39)-amide an antagonist at the glucagon-like peptide 1-(7–36)-amide receptor of insulin-secreting beta-cells. J Biol Chem. 1993;268: 19650–5. 8396143

[38] Lupi R, Mancarella R, Del Guerra S, Bugliani M, Del Prato S, Boggi U, et al Effects of exendin-4 on islets from type 2 diabetes patients. Diabetes Obes Metab. 2008;10: 515–9. 10.1111/j.1463-1326.2007.00838.x 18201204

[39] Park YJ, Ao Z, Kieffer TJ, Chen H, Safikhan N, Thompson DM, et al The glucagon-like peptide-1 receptor agonist exenatide restores impaired pro-islet amyloid polypeptide processing in cultured human islets: implications in type 2 diabetes and islet transplantation. Diabetologia. 2013;56: 508–19. 10.1007/s00125-012-2802-z 23262664

[40] Cechin SR, Perez-Alvarez I, Fenjves E, Molano RD, Pileggi A, Berggren P-O, et al Anti-Inflammatory Properties of Exenatide in Human Pancreatic Islets. Cell Transplantation. 2012;21: 633–648. 10.3727/096368911X576027 21669040

[41] Kim W, Egan JM. The role of incretins in glucose homeostasis and diabetes treatment. Pharmacol Rev. 2008;60: 470–512. 10.1124/pr.108.000604 19074620 PMC2696340

[42] Garber AJ. Incretin effects on beta-cell function, replication, and mass: the human perspective. Diabetes Care. 2011;34 Suppl 2: S258–63. 10.2337/dc11-s230 21525465 PMC3632189

[43] Drucker DJ. Incretin action in the pancreas: potential promise, possible perils, and pathological pitfalls. Diabetes. 2013;62: 3316–23. 10.2337/db13-0822 23818527 PMC3781450

[44] Bentsi-Barnes K, Doyle ME, Abad D, Kandeel F, Al-Abdullah I. Detailed protocol for evaluation of dynamic perifusion of human islets to assess beta-cell function. Islets. 2011;3: 284–90. 21811103 10.4161/isl.3.5.15938 PMC3219161

[45] Abdul-Ghani MA, DeFronzo RA. Pathophysiology of prediabetes. Curr Diab Rep. 2009;9: 193–9. 19490820 10.1007/s11892-009-0032-7

[46] ADA. Classification and Diagnosis of Diabetes. Diabetes Care. 2016;39: S13–S22. 26696675 10.2337/dc16-S005

[47] Bergman RN, Phillips LS, Cobelli C. Physiologic evaluation of factors controlling glucose tolerance in man: measurement of insulin sensitivity and beta-cell glucose sensitivity from the response to intravenous glucose. J Clin Invest. 1981;68: 1456–67. 7033284 10.1172/JCI110398 PMC370948

[48] Deeds MC, Anderson JM, Armstrong AS, Gastineau DA, Hiddinga HJ, Jahangir A, et al Single dose streptozotocin-induced diabetes: considerations for study design in islet transplantation models. Lab Anim. 2011;45: 131–40. 10.1258/la.2010.010090 21478271 PMC3917305

